# War in Ukraine: a neurosurgical perspective

**DOI:** 10.1007/s00701-022-05388-3

**Published:** 2022-10-20

**Authors:** Daniel Dubinski, Volodymyr Kolesnyk

**Affiliations:** 1 Department of Neurosurgery, Rostock University Medical Center, Rostock, Germany; 2 Department of Neurosurgery, Central City Clinical Hospital, Sumy, Ukraine

**Keywords:** Ukraine, War, Neurosurgery, Neurotrauma, Wartime medicine, War-related injuries

## Abstract

**Background:**

The ongoing war in Ukraine leads to the destruction of critical infrastructure and the displacement of millions of civilians while the necessity for neurosurgical care has increased tremendously. The consequences of this armed conflict on the practice of neurosurgery are uncertain to date.

**Methods:**

A cloud-based questionnaire including 10 single- and multiple-choice questions was sent through the email distribution list of the *Ukrainian Neurosurgical Society* and the *Association of Neurosurgeons of Ukraine*.

**Results:**

Between June 2022 and July 2022, a link to the online survey was distributed to a total of 134 (100%) departments of neurosurgery across Ukraine. After 21 days of being available, a total of 96 questionnaires (72%) returned.

**Conclusions:**

The survey highlights the field of activity as well as the severe impact on professional and personal life of Ukrainian neurosurgeons during the ongoing war.

## Introduction

At the time of writing this paper, an unknown number of civilians have already lost their lives due to the armed raid against Ukraine which started on February 24, 2022. The number of wounded among civilians and military personal cannot yet be estimated. By July 2022, nearly 8 million people have been displaced and more than 6.6 million escaped the country [8].

This war hits a country whose health care system is still far away from Western standards due to insufficient governmental funding, extensive centralization, and generally outdated equipment [5]. For instance, there is great shortage of operating microscopes which are indispensable for the surgical care of peripheral nerve injuries [5].

In this war, the indiscriminate attacks on civilian infrastructure also damaged health care facilities such as the Central City Hospital in Vuhledar with at least three casualties, thereby further deteriorating the care of the wounded [9]. Shrapnel wounds, bone fractures, and burns are the predominant injuries in surviving victims after bomb raid and missile attacks [6].

Recently, Loskutov et al. reported on the special challenges for medical personnel, as during air alarms, the ICU staff are usually not able to seek shelter. To this adds the lack of blood products, drugs, and equipment and the burdensome handling of the injured in basements and shelters [2].

We have tried to obtain some direct information about the impact of the current hostilities in Ukraine on neurosurgical activities and work demand.

## Methods

The authors developed a 10-point questionnaire including single- and multiple-choice questions and sent it out to the 134 Ukrainian neurosurgical departments as listed by the two professional societies. The survey was sent via email on June 30, 2022, using the cloud-based SurveyMonkey® and closed after 21 days.

The questions submitted concerned the type of neurosurgical facilities and the impact of the hostilities on the infrastructure, the neurosurgical activity, and workload (Table 1). Since the questionnaire does not involve human subjects or patient history, a vote from an ethics committee was not necessary. Each recipient of the survey was requested to respond only once and was unable to edit the response once submitted. With regard to the type of study, statistical analysis appeared inappropriate.

**Table 1:** Overview of the survey results

Question asked *N* = 96					
Workplace characterization	What hospital do you work at?	Public hospital	Privat hospital		
		95 (99%)	1 (1%)		
	What diagnostic equipment do you have at your disposal?	CT scan	MRI	Angiography	All of the above
		42 (44%)	7 (7%)	2 (2%)	45 (47%)
	What is the distance to the next neurosurgery?	< 100 km	< 500 km	> 500 km	
		72 (75%)	22 (23%)	2 (2)	
Influence of war on infrastructure	Was your clinic under attack?	Yes	No		
		8 (8%)	88 (92%)		
	Was the electricity supply uninterrupted?	Yes	No		
		66 (69%)	30 (31%)		
	Was the sterilization working continuously?	Yes	No		
		79 (83%)	16 (17%)		
Neurosurgical activity	Have you performed surgeries outside the neurosurgical spectrum?	Yes	No		
		5*5* (57%)	41 (43%)		
	Did your staff (doctors/nurses) suffer military injuries ?	Yes	No		
		7 (7%)	89 (93%)		
	Most of the consequences of war trauma were	Spinal injury	Traumatic brain injury		
		21 (22%)	75 (78%)		
	Are you in contact with Russian neurosurgeons?	Yes	No	I would not like to answer	
		2 (2%)	90 (94%)	4 (4%)	

## Results

A total of 96 neurosurgeons responded to our survey. Assuming that no multiple answers originated from the same clinic, a representative response rate of 72% (96/134) was achieved.

### Workplace characterization

From the 96 participants, 95 (99%) work in a governmental hospital and a single one (1%) in a private hospital. Regarding the diagnostic imaging, 36% of the participants report that they have all of the radiological diagnostic tools at their disposal. Equipped only with a CT scan were 44%, MRI only 7%, and angiography only 2%.

With regard to the geographical distribution of neurosurgical facilities, 75% of the participants reported a distance of less than 100 km (62 miles) to the next neurosurgical unit, 23% indicated a distance of less than 500 km, and in 2%, the next neurosurgical department is more than 500 km (311 miles) away.

### Influence of the war on infrastructure

Eight participants reported on at least one attack on their clinic. In seven units, staff members had already suffered war-related injuries. Transient interruption of power supply was reported by 31% of the respondents. In 17% of hospitals, the sterilization facility failed, at least temporarily.

### Neurosurgical activity

Fifty-seven percent of the participants have already been forced to perform surgery outside their own specialty. Seventy-eight percent reported TBI as the predominant type of war-related trauma to the central nervous system whereas 22% reported on the predominance of spinal cord injuries. When asked about any maintained contact with Russian neurosurgical colleagues, 90/96 participants answered “no,” two responded with “yes,” and four respondents refuted an answer.

## Discussion

The present data reveal the profound impact of an ongoing war on neurosurgical facilities, workload, and personal life of staff members. Neurosurgeons from Ukraine were in part personally harmed by the attacks, forced to work under adverse conditions, and performed surgeries outside their common neurosurgical spectrum.

The data obtained in our survey are inevitably scarce due to secrecy obligations in wartime. But they reaffirm the fact that this war has hit a country whose health care system had to struggle against governmental underfunding and poor supply with equipment even before. With regard to neurosurgical facilities, there is considerable deficiency of modern diagnostic tools such as MR imaging systems and neuroradiological intervention devices such as angiography. In 2013, MacKenbach et al. found that Ukraine had the lowest health policy performance score of all 43 European countries analyzed [3]. Their statement is confirmed by the reported distances between the neurosurgical units. It is further underlined by the number of 2.99 physicians and 7.5 hospital beds per 1000 residents in Ukraine, as reported in 2014 [10]. The situation is still deteriorating by indiscriminate attacks on civil infrastructure including hospitals, e.g., causing damage to power supply and threatening the lives of medical personnel.

While 8% of the responding units had suffered from a military attack with injuries to their personnel, the WHO recorded in Ukraine a total of 344 attacks that damaged health care facilities between February 24 and July 23, 2022 [9]. This included attacks causing casualties among medical personnel. As a tragic fact, attacks on health care facilities including fatalities among staff members are not rare in war zones as reported by the NGO *Doctors Without Borders *[8]. The same is true for interruption of power supply and sterilization facilities [4].

Concerning the patterns of injury, the reported predominance of TBI in the present study is in line with published data of the US military during US combat operations between 2002 and 2016 where craniotomy/craniectomy was the most frequent procedure (62%) [7].

The majority of respondents (*N* = 55/57%) were forced to perform surgery outside their neurosurgical spectrum. This fact indirectly reflects the undersupply of Ukrainian medical personnel as well as a growing patient load caused by combat.

Meanwhile, most (94%) of the Ukrainian respondents cautiously indicated that contact with Russian neurosurgical colleagues has been interrupted. This reveals another tragedy in view of the deep common roots of Ukrainian and Russian neurosurgery that have until now withstood the political circumstances as proved by the participation of Ukrainian neurosurgeons at the 9th congress of Russian Neurosurgeons in 2021 [1]. There can be no general conclusion under these, still developing events. Rather, this article represents the information available at the present time on the everyday situation of Ukrainian neurosurgeons who are exposed to wartime conditions. The ultimate consequences of the ongoing war for Ukrainian neurosurgery remain undetermined and could be further explored in future surveys.

### Limitations

The biggest bias of our survey is the anonymity of the respondents, which is attributed to secrecy demands. Hence, we cannot with certainty exclude multiple or even false responses.

## Conclusions

War is the most abhorrent act upon humanity. Ukrainian neurosurgical departments operate in wartime under severe restrictions of human and technical resources. Attention should be paid to direct medical supply to unsupported and geographically isolated neurosurgical departments across Ukraine.

## Abbreviations

TBI: Traumatic brain injury
CT scan: Computed tomography scan
MRI: Magnetic resonance imaging
WHO: World Health Organization
NGO: Non-governmental organization
